# Measuring individuals' response quality in self-administered psychological tests: an introduction to Gendre's functional method

**DOI:** 10.3389/fpsyg.2015.00629

**Published:** 2015-05-15

**Authors:** Marc Dupuis, Emanuele Meier, Roland Capel, Francis Gendre

**Affiliations:** Institute of Psychology, University of LausanneLausanne, Switzerland

**Keywords:** functional method, exploratory factor analysis, psychometrics, response reliability, response validity, self-rated questionnaires

## Abstract

The *functional method* is a new test theory using a new scoring method that assumes complexity in test structure, and thus takes into account every correlation between factors and items. The main specificity of the functional method is to model test scores by multiple regression instead of estimating them by using simplistic sums of points. In order to proceed, the functional method requires the creation of hyperspherical measurement space, in which item responses are expressed by their correlation with orthogonal factors. This method has three main qualities. First, measures are expressed in the absolute metric of correlations; therefore, items, scales and persons are expressed in the same measurement space using the same single metric. Second, factors are systematically orthogonal and without errors, which is optimal in order to predict other outcomes. Such predictions can be performed to estimate how one would answer to other tests, or even to model one's response strategy if it was perfectly coherent. Third, the functional method provides measures of individuals' response validity (i.e., control indices). Herein, we propose a standard procedure in order to identify whether test results are interpretable and to exclude invalid results caused by various response biases based on control indices.

## Introduction

For about a century, psychological testing has become an important aspect of psychologists' activity with an ever-growing importance. Nonetheless, despite formidable developments in psychometrics during the last several decades, most clinical and scientific practices covering psychological assessment continue to refer to the nearly unchanged method inherited from classic test theory (CTT). Many critics have been raised against the classic method: In particular, it is too often assumed that items have no second loadings, and that each item has the same weight, which is optimal for confirmatory factor analysis, but it also leads to a loss in reliability. Furthermore, though CTT assumes requirements in test validity (e.g., satisfactory reliability, concurrent validity), few requirements have been formulated concerning individuals' ways of responding to self-administered questionnaires.

The functional method is a new method that improves test reliability and provides indices of one's response validity to self-administered tests. First, three major issues of classic testing are examined; then item response theory (IRT) is presented as the main alternative to CTT. Last, the functional method is presented with a focus on how it deals with these problems and compared with CTT and IRT.

### The problem of response intrinsic quality

It is well known that psychological tests can be biased both intentionally and unintentionally; this can invalidate one's test results, but can even invalidate an entire test validation (Caldwell-Andrews et al., 2000; Marshall et al., 2005). According to Jackson and Messick (1958), two kinds of response biases must be differentiated: Response sets, that are temporary reactions to a contextual explicit or implicit expectation must be distinguished from response styles, which covers permanent—and “less” intentional—response biases.

According to Cronbach (1946), invalid response sets may include “aware” response sets that can invalidate test profiles, such as item omission or guessing what is the most desirable answer. In addition, acquiescence and extreme responding were also identified as highly detrimental response biases (Cronbach, 1946; Harzing, 2006). From an environmental perspective, Flaugher (1978) depicted various sources of differences in response patterns related to gender, ethnicity, or culture. He also highlighted the impact of context, particularly, what is at stake with the assessment, which can lead to overestimating or faking some dimensions, either in self-description (Furnham and Henderson, 1982) or in describing close friends or relatives (Pedregon et al., 2012). In 1968, Mischel observed that the correlations between assessments of the same constructs in different situations were rarely higher than 0.30 (Patry, 2011). Finally, careless and deviant responding could include either temporal or stable patterns that also impact the validity of one's results to self-rated questionnaires (Paulhus, 1991).

An entire field of psychological science is dedicated to the development of techniques to detect and minimize response biases. Yet, a first issue in classic testing is that such biases are rarely investigated or controlled in clinical practice. Some of these biases can be detected easily to exclude invalid test results.

### The problem of arbitrary and relative metrics

Arbitrary metrics present another issue in testing. Indeed, as with any measures, test scores are uninterpretable without external references. Generally, psychometric references are normative sample means and standard deviations. When tests scores must be meaningful, a nonarbitrary solution consists of standardizing these scores. Nevertheless, standardization does not simply make a metric nonarbitrary; rather, it makes the metric more relative and dependent on the quality of sampling (Blanton and Jaccard, 2006b). Thus, standardized scores are meaningful for understanding how individuals from the reference group responded, but not for how a given individual assessed responds. Overall, norm-referenced measures depend on the representativeness of the normative sample. Assuming that some response biases are natural, such a sample either includes biased data (and is thus problematic) or is not very representative (Embretson, 1996, 2006).

According to Blanton and Jaccard (2006a), many researchers have no interest in characterizing participants, so there has been no effort to define what some high values in a dimension really mean. Nevertheless, researchers' disinterest does not alleviate the concern of how to communicate test results clearly to test respondents in clinical practice, which is a major issue of CTT-based assessment.

### The problem of simplicity

The last and most important issue is related to the following dilemma: How simple could a test be in order to be both valid and user-friendly? Indeed, in the case of a multidimensional test, “pure” items are largely preferred to items related to more than one factor. A defendable reason is that factor structures with too many cross-loadings are unidentifiable, making them unsuited to confirmatory factor analysis. Still, such statistical arguments have been overused. For example, Nunnally (1967) suggested eliminating items with cross-loadings and excluding items where loadings are lower than 0.30 because they reduce the model fit. Such a recommendation implies that a test should be as clean as it can be, no matter how simplistic and unrepresentative it becomes, in order to obtain satisfactory fit indices from confirmatory factor analyses. Yet, such methodological concerns are barely questioned; even more striking, they are still taught as the best practices (e.g., Costello and Osborne, 2005). A related issue is that “pure” scales are more likely to provide high internal consistencies or split-half reliabilities, which reinforces the deviant practice of test purification. As a consequence, such high values for reliability indices are the causal effect of a test's purity rather than an evidence of its validity. Another well-accepted yet detrimental consequence is that pure tests are more transparent, making latent traits so recognizable that people can optimize their responses with a relative ease.

Of those three issues, each of the two first is nested within the latter; and all of them have the assumption of simplicity in common. The quest for an understandable scale leads to purify either test dimensions or test metrics, which makes them clearer to the practitioner and to the respondent, as well, therefore more easily biasing tests on intentional and unconscious levels. On the other hand, assuming test complexity instead might be a better way to deal with those issues. Indeed, computers have radically changed psychometrics, so that some difficult mathematical operations can now be performed automatically, quickly and at no cost. Such a technical background provides new solutions to the aforementioned issues.

### Item response theory

Because of the limitations inherent in using CTT, critics have raised many questions regarding the relativity of norm-referenced measures. Thus, IRT was conceived to address this specific concern. In a now-famous paper, Embretson (1996) listed the differences in the rules of measurement between CTT and IRT (cf. Embretson and Hershberger, 2012); five of these are of particular interest:

1. The first difference concerns the standard error of measurement. In CTT, the standard error of measurement is assumed to be consistent across scores, but varies across populations. By contrast, in IRT, the standard error of measurement varies across scores, but is consistent across populations.
2. The second main difference concerns the relationship between the length of a test and its reliability. Indeed, a bold assumption of CTT is that the longer a test is, the more reliable it is. On the contrary, IRT-based adaptive testing eventually makes short tests as reliable as long ones.
3. The third difference addresses comparability in different test scores. In CTT, test parallelism is required to compare test scores across different forms; in IRT, scores in different tests can be compared even when the tests are considered nonparallel.
4. The fourth difference concerns the assessment of the items' characteristics. In CTT, item properties must be assessed using representative samples, while IRT can also rely on data from non-representative samples to achieve the same goal.
5. The last difference concerns the metric and meaning of the scores. As stated, the metric used to express scores in CTT is norm-referenced (i.e., standard scores or percentiles), which has limitations (i.e., relative metric, questionable sampling, etc.). In addition, CTT also is likely to use raw scores (i.e., integer values) rather than a continuous scale. In IRT, the trait scores are expressed in the same metric as the items (i.e., in logits). Such a common metric makes it possible to provide a clear and probabilistic interpretation: An individual whose score falls below an item score is simply more likely to fail in responding to a question or to give the answer coded as the lowest value. In this “absolute” metric, scores have a meaning that is directly referenced to the items, so that scores referring to some norms are no longer necessary. In addition, it is feasible to compare scores in different tests when calibrated together because there is an absolute common metric.

By expressing individuals (i.e., their scores) and items in a common metric, IRT has two advantages. First, diagnosing misfit between items and persons is possible, which is of great interest in detecting unusual response patterns. Second, detecting differences in item functioning is also possible, which can be used to calibrate a test consisting of the most relevant items.

IRT (including its generalized forms) is the only model commonly used to measure psychological constructs and express them in an absolute metric. Yet, no natural metric is associated with IRT models, which implies that both zero- and scale-metrics require specification. In other words, though IRT is concerned with the problem of metrics' arbitrariness and relativeness, it deals with the relativeness but it eventually expresses test scores in arbitrary metrics. In addition, IRT models rely on different bold assumptions that do not apply to every type of data (cf. Magno, 2009). Furthermore, IRT was initially developed as an approach for unidimensional constructs rather than multidimensional constructs, and for dichotomous items rather than polytomous ones. Consequently, unidimensional IRT models are considered more suitable for Likert-type data than multidimensional ones (Maydeu-Olivares, 2005). This could be considered IRTs' most important limitation.

IRT provides some compelling alternatives to counter CTT limitations, and is a well-implemented approach in the fields of intelligence and education psychology (Reise and Henson, 2003). Yet, several authors concluded that CTT and IRT led to very similar results in most cases (Nunnally, 1967; Fan, 1998; Willse and Goodman, 2008), including with self-administered tests (MacDonald and Paunonen, 2002). Thus, learning IRT appears to be an important investment in time that differs from CTT only slightly, which could explain why fields such as personality psychology or vocational psychology have shown little interest in this approach (Reise and Henson, 2000, 2003). By contrast, an approach theorized to deal with multidimensional data measured by polytomous items might be more promising for such fields of research.

## The functional method

The functional method is a new theory that consists of a new scoring method developed by Gendre (1985) and applied by Capel (2009) to different psychological fields, including personality assessment, vocational guidance, and values assessment (Gendre et al., 2011, 2012; Gendre and Capel, 2013). So far, the functional method has been clarified and introduced in the French-speaking literature (Gendre et al., 2002, 2007), but has yet to be introduced in English to an international audience.

### Main principles

Historically, as the most commonly-used theories, CTT and IRT were conceived for use with aptitude tests but later applied to attitudinal measures. However, tests measuring attitudes differ greatly from aptitude tests in the construction of the items and in the process of responding to them, as well. Indeed, aptitude tests rely on simple and objective items (with objectively correct and incorrect answers), and the response process is assumed to be attributable to only unidimensional factors. Conversely, attitude tests rely on subjective items expressed on a Likert-type scale, and they imply comparing the meaning of the item to the global image of oneself. Therefore, attitude tests are non-metric and multivariate. Unlike CTT and IRT, the functional method theory (FMT) was specifically conceived for multidimensional constructs measured in ordinal Likert-type scales; it starts with the insight that everything in a psychological test is carried in item responses, including the process of responding, which is considered a latent variable. FMT is a test theory that is:

– Global: tests are taken as complete and coherent constructs, not as a juxtaposition of differentiated scales;
– Multivariate: items are considered as complex and multidimensional;
– Efficient: it consists of different optimal multivariate methods: exploratory factor analysis (item characterization), nonmetric multidimensional scaling (response metrication), and multiple regression (analysis of response strategy);
– Complete: it provides absolute comparable scores (within individuals) that can eventually be standardized in order to compare between individuals; this leads to two complementary perspectives: how one perceives oneself, and how measures up compared to a specific sample;
– Economical: it uses any available information in order to provide the most reliable measures; such measures can result from rational (i.e., expert judgment), statistical (i.e., item factor analysis) and/or empirical (i.e., items' validations) methods of test construction;
– Cautious: it considers response entropy in order to determine if one's response pattern produces either valid or unusable results.

The functional method mixes different specificities from CTT and IRT, with some new benefits. FMT was specifically conceived for multidimensional constructs, expressing them in an absolute metric, but also using every item to estimate every latent trait. Therefore, FMT is based on three theoretical principles that neither CTT nor IRT assume. First, since both complexity and purification are opposing sources of error, one must decide which one is more acceptable to deal with; thus, FMT assumes that complexity is preferable. Indeed, while CTT considers most minor cross-loadings as negligible or as detrimental information, FMT takes each of them into account in order to improve measurement precision. Second, given that classic test measures rely on approximated scores, FMT assumes that test measures may rely on modeled scores instead, meaning that scores are the results of multiple regression modeling. Third, much like IRT, FMT assumes that items, individuals, and factors can be expressed as vectors, which implies that all axioms or theorems developed in vector geometry are applicable.

### Technical procedure

Technically, the functional method requires the creation of a measurement space that is absolute and exhaustive, wherein items, persons, and other outcomes can be expressed in the same metric. This measurement space must take the form of an orthonormal unit hypersphere where items are expressed by its radius vectors. Thus, the vector coordinates correspond to items' characteristics, which consist of their absolute loadings (without error terms) on the fundamental dimensions of the measurement space. The creation of such a measurement space is simple, and differs slightly from the creation of CTT measurement space, although both result from factor analysis. Regarding FMT, factors must be extracted by principal component analysis (PCA)^1^ followed by Varimax rotations on all or some of the retained components to ensure a better interpretation of the measurement space. Nevertheless, FMT is based on multiple iterations of the PCA. This first PCA results in a loading matrix, which requires further transformations. Thus, the next step consists of performing a PCA on the loading matrix onto which lines have been normed, of extracting factor scores from this PCA, and of conducting another PCA on extracted factor scores. Then, the PCA can be reiterated on the resulting factor scores as many times as required—until there is strict orthogonality between factors and until each item's norm is equal to 1, indicating a communality of 100%. This requires forcing the successive PCA to extract the same number of factors as the first PCA, and reiterating Varimax rotation.

What results from the successive transformations of the loading matrices is a matrix that expresses the characteristics of each item in a same metric: Each matrix line is a vectorial expression of an item and each item is a radius of a same hypersphere, in which the dimensions are the columns (i.e., the factors). This matrix is called “K,” the *matrix of item characteristics* (consisting of *k* items × *i* factors). More than a simple matrix, K is the final measurement space in which responses are represented. For computational purposes, the K matrix can be transformed into “KZ” by transforming its columns into z-scores.

Beside the measurement space is the response space, a vector containing one's response to each item, which is generally expressed on a Likert-type scale, but can only be considered an ordinal scale from a mathematical point of view; this scale must be transformed into a metric scale before being used. In a universe composed of *k* items, this metricised vector is expressed: $\vec{x_{k}}$. It models response strategy^2^ regarding the entire questionnaire. Thereby, each item response contributes to the response strategy. More interesting is the possibility to model the response strategy by applying it to the test's dimensions. This can be obtained by calculating $\vec{s}$, the *vector of strategy*, which is composed of the correlations between $\vec{x_{k}}$ and each column of KZ:

$$Vector\;of\;strategy=\vec{s}=\left(\begin{array}{c} S_{1}\\ S_{2}\\ S_{3}\\ \ldots \\ S_{i} \end{array}\right)\;=\;\left(\begin{array}{c} cor\left(\vec{x_{k}},\;\vec{KZ_{1}}\right)\;\\ cor\left(\vec{x_{k}},\;\vec{KZ_{2}}\right)\\ cor\left(\vec{x_{k}},\;\vec{KZ_{3}}\right)\\ \ldots \\ cor\left(\vec{x_{k}},\;\vec{KZ_{i}}\right) \end{array}\right)$$

In FMT, $\vec{s}$ is the canonical form of non-normed factor scores, which can eventually be normed and expressed in other metrics. In its canonical form, response strategy can be analyzed.

## Functional control indices

One of the main interests in FMT is the way that it deals with the problem of intrinsic response quality. Indeed, FMT takes advantage of the creation of *control indices* that enable the detection of problematic response strategies. Some of those indices are specific to FMT; they can be used to determine whether test results are valid and interpretable. Others are used to highlight specific biases and can identify some sources of error (see Table 1).

**Table 1:** **Functional control indices and their definitions**.

**Control index**	**Definition**
Response coherence	A measure of how coherent and predictable the response strategy is. High values in coherence can suggest that one is faking one's answers, while low values in coherence indicate that one is very atypical in one's response pattern, that one completed the questionnaire carelessly, or that one had problems in understanding the items
Response reliability	A measure of how stable the response strategy is, obtained after applying the bisection method. A low value in reliability can be interpreted as a lack of application or problems in understanding the items, and negative values surely highlight random response patterns
Response mean	A measure of response centrality. In tests with reverse-coded items, high means can indicate that the participant agreed with most items regardless of their meaning, highlighting an acquiescence bias
Response variability	A measure of response dispersion. Response sets with little variability indicate that the participant gave little information due to either extreme or central answers. This also invalidates the results (which are always based on response variance)
Response modality	A measure of how often the modal answer is chosen, using Cohen's weighted kappa. High values in modality can suggest that the subject attempted to describe himself or herself as a very banal person. More interestingly, very negative values in modality highlight that the participant might have made a mistake by reporting reversed answers
Response normativity	A measure of how much a response set fits general response tendencies, using the correlation between the participant's answers and the means of each item. This measure's main use is to detect reversed answers, but it is also sensitive to socially desirable responding
Response positivity and negativity	Measures of how much both positive and negative aspects of personality have been accentuated in one's self-description. Such measures are interesting in order to detect unbalanced self-descriptions: depending on the context (e.g., applying for a job), people can overrate positive dimensions and underrate negative dimensions, which can be highlighted by calculating the difference between positivity and negativity

### Response coherence

Given that item characteristics are orthogonal and standardized in the KZ matrix, the correlations expressed by $\vec{s}$ are also the beta weights of a multiple regression model that can predict a person's response. Such conditions legitimate the calculation of a multiple correlation index that indicates the degree of fit between the person's response and the item characteristics; this is why it is named *response coherence*. A high coherence means that the individual was able to describe himself or herself in a conceptual framework that is shared by the individual and the test itself (i.e., other individuals whose responses were part of the test calibration); indeed, a high coherence implies that the responses given consist of a complex but still predictable set of information. On the other hand, a low coherence means that the responses given are not very predictable using all of the other ones.

Mathematically, the coherence is equivalent to the norm of the vector of strategy:

$$Response\;coherence=\left\|\vec{s}\right\|=\sqrt{\sum_{1}^{i}\left[cor{\left(\vec{x_{k}},\vec{KZ_{i}}\right)}^{2}\right]}$$

Consequently, the vector of strategy can be normed by dividing it by the coherence, providing standard z-scores for each factor:

$$Normed\;vector\;of\;strategy=\frac{\vec{s}}{\left\|\vec{s}\right\|}=\left(\begin{array}{c} z_{1}\\ z_{2}\\ z_{3}\\ \ldots \\ z_{i} \end{array}\right)$$

This point has important implications for subject comparison: Consistent with both CTT and IRT, it means that extreme responses are incoherent. Reciprocally, low coherence makes the standard scores extreme. Over all, the mathematical relation between standard factor scores and response coherence highlights that unsatisfactory coherence makes the standard scores unreliable. This is why such an index should be checked even before considering the interpretation of test results. Furthermore, another important mathematical quality of the response coherence is that it corresponds to the square root of the communality of the vector of strategy. This implies that such an index refers to a coefficient of determination, thereby indicating how predictable one's vector response is. This means that the coherence is a direct indicator of the percentage of interpretable and usable information in one's responses.

According to Capel (2009), high values in coherence could be interpreted as a sign of response refinement due to socially desirable responding, or even faking. Such phenomena can be observed in selection contexts (Bagby and Marshall, 2003), and are as problematic as low coherence. Because a statistical model is not human, people are unlikely to have a response coherence of 1, so such cases indicate that participants gave the description of an expected model rather than of themselves. Capel (2009) stated that low values in response coherence could result from a lack of maturity and are likely found in adolescent participants; this is consistent with Soto et al. (2008) results with another approach of test results coherence.

### Response reliability

Another important measure of one's response quality is *response reliability*. Response reliability is an unusual application of a well-known technique used in test validation, namely the bisection method. Usually, the bisection method is used to calculate the split-half reliability of a test among a sample of subjects' answers; calculating split-half reliability in one only person's responses is of little relevance and is quite unfeasible in CTT. Yet, Drewes (2000) highlighted that reliability of a measurement can be modeled. Thereby, FMT's purpose is to calculate split-half reliability based on one's modeled response. Thus, two vectors of strategy ($\vec{s^{\prime }}$ and $\vec{s^{\prime \prime }}$) can be obtained from two halves of the original questionnaire completed by one individual; the correlation between those two vectors is a first approximation of the person's response reliability. Still, conforming to its classical use, the bisection method underestimates the reliability due to the number of items taken into account by the half tests. A first solution is to calculate the exact split-half reliability. Because Cronbach's alpha is not calculable in FMT (given that every item is related to every factor); another possibility is to calculate the mean correlation between each parallel form, which is a feasible but far too laborious solution. As a remaining possibility, the reliability can be adjusted using the Spearman-Brown correction formula:

$$\begin{array}{l} Corrected\;response\;reliability=r^{SB}\\ \;\;\;\;\;\;\;\;\;\;\;\;\;\;\;\;\;\;\;\;\;\;\;\;\;\;\;\;\;\;\;\;\;\;\;\;\;\;\;\;\;\;\;=\;\frac{2*cor\left[\left(\begin{array}{l} S_{1}^{{}^{\prime }}\\ S_{2}^{\prime }\\ S_{3}^{\prime }\\ \ldots \\ S_{i}^{\prime } \end{array}\right),\left(\begin{array}{l} S_{1}^{\prime \prime }\\ S_{2}^{\prime \prime }\\ S_{3}^{\prime \prime }\\ \ldots \\ S_{i}^{\prime \prime } \end{array}\right)\right]}{1+cor\;\left[\left(\begin{array}{l} S_{1}^{{}^{\prime }}\\ S_{2}^{\prime }\\ S_{3}^{\prime }\\ \ldots \\ S_{i}^{\prime } \end{array}\right),\;\left(\begin{array}{l} S_{1}^{\prime \prime }\\ S_{2}^{\prime \prime }\\ S_{3}^{\prime \prime }\\ \ldots \\ S_{i}^{\prime \prime } \end{array}\right)\right]\;}\; \end{array}$$

Response reliability is of great importance in interpreting test results. A high response reliability indicates that one completed the questionnaire with care, that one was able to understand the meaning of the items, and that one was stable in the manner s/he responded throughout the test. On the other hand, a low response reliability means that the person had problems in responding, consisting either of a lack of care, a lack of comprehension, or a lack of stability in responding (e.g., due to fatigue). Indeed, one of those three issues is enough to make test results completely unreliable. In addition, unreliable responses also are likely to lower response coherence, which makes them totally unusable. Thus, response reliability should always be checked before interpreting tests results.

### Response level and variability

Though response coherence and response reliability are the main control indices for considering whether a set of test responses is valid enough to be interpreted, more information is required in order to identify what makes the results invalid or deceptive. Therefore, some additional indices are of great use in order to confirm the existence of response biases. First, two simplistic but useful indices may be proposed: *response level* and *response variability*. Neither of these is specific to FMT, and both can be easily calculated (also in CTT). Response level consists of the mean of the responses given to each item; it must be calculated before recoding reverse-coded items. Consistent with information theory, response level has to be balanced in order to maximize information; in other words, response level should not be too close to the extreme ends of the Likert-type scale used. Moreover, response level carries psychological meaning in self-rated scales; for example, it can be used to estimate overall motivation in responses to a test of vocational interests (Gendre et al., 2012). In addition, response level can be normed in order to specifically detect acquiescence or opposition biases.

Response variability consists in response standard deviation. Also consistent with information theory, response variability has to be balanced. Null values in variability mean that the person has used the same answer thorough the questionnaire. Opposition may cause this, formally invalidating the results: Such a response pattern leads to no information. While in CTT, results are still calculable and may be wrongfully interpreted, in FMT, where the scoring is correlation-based, results are simply not calculable. Similarly, a low value in response variability is the sign of a lack of usable information, suggesting that one was reluctant to provide information about oneself. By contrast, high values in response variability are generally preferable, but can consist of extreme responding biases as well.

### Response modality and normativity

An important issue in psychological assessment is *socially desirable responding* (Paulhus, 1991, 2002). Depending on the context and the content of the test, socially desirable responding can lead either to extreme or banal responses (i.e., dissimulating undesirable traits). Thus, two kinds of indices can be used in order to gain some insight on such phenomena, namely *response modality* and *response normativity*. Response modality refers to one's tendency to report modal answers to items. Different formulas can be proposed in order to estimate response modality. According to Capel (2009) response modality can be measured by the mean quotient between the proportion of people who gave the same answer to the item and the proportion of people who answered by the modal answer:

$$Response\;modality\;(Capel^{\prime }s\;formula)=\;\frac{\sum \frac{p\left(X_{k}\right)}{p\left(mode\left(X_{k}\right)\right)}}{k}$$

Capel's original formula resulted in a value that could vary from 0 to 1. Nonetheless, a preferable possibility is to use Cohen's weighted kappa to measure how much one's answers converge with each modal answer, providing a correlational index that could be transformed into the percentage of variance explained by modal responding.

A second index related to desirable responding is response normativity. Response normativity consists of the correlation between one's answers and the means of each item. This index is less relevant for measuring how often modal answers are given than response modality; instead, it focuses on the global fit with mean answers. In other words, response normativity measures whether the person answered by high values where people generally do and by low values where people generally do, or whether the person did not. Furr (2008) and Leary and Allen (2011a,b) proposed conceptualizing self-description as a function of two tendencies: a normative presentation fitting the norm group, and a distinctive description that covers differences with the norm group. In these terms, response normativity is a measure of the normative part within one participant's self-description, while response modality is a measure of the distinctive part within his or her self-description (i.e., the higher the response modality is, the less distinctive one's self-presentation is).

Response modality and response normativity are highly correlated and should be interpreted together. High values in response modality imply having chosen many modal answers, which is a form of both banality and social conformity. Regarding response normativity, high values mean that one's response is highly correlated with item means; in vectorial terms, this means that one's vector of strategy and peoples' mean vector of strategy have the same direction (i.e., there is only a small angle between both vectors), which can be the case even if the response modality is low. Concerning both indices, very low or even negative values in both response modality and normativity suggest a problematic response strategy. It is compatible with acceptable response coherence and response reliability, but can results from reporting reverse-scored answers by mistake; according to Capel (2009), such mistakes can be found among 1–2% of personality questionnaires. In that case, the only solution is to ask what the person meant by answering the way that s/he did.

### Response positivity and negativity

Finally, two functional indices are used in order to investigate socially desirable responding: *response positivity* and *negativity*. In order to calculate them, most positive and negative items according to individuals' ratings are selected from the KZ matrix in order to create two matrices respectively related to positive or negative items. Then, new vectors of strategy can be calculated, based only on the selected positive or negative items: one related to positive aspects, and another related to negative aspects. Last, response positivity corresponds to the sum of the product of the vector of strategy and the vector specific to positive contents:

$$\begin{array}{l} Response\;positivity=\frac{\vec{s}}{\left\|\vec{s}\right\|}\times \frac{{\vec{s}}^{+}}{\left\|{\vec{s}}^{+}\right\|}\\ \;=\sum_{1}^{i}\left[\left(\begin{array}{c} z_{1}\\ z_{2}\\ z_{3}\\ \ldots \\ z_{i} \end{array}\right)\times \left(\begin{array}{c} z_{1}^{+}\\ z_{2}^{+}\\ z_{3}^{+}\\ \ldots \\ z_{i}^{+} \end{array}\right)\right] \end{array}$$

In the same way, response negativity is the sum of the product of the vector of strategy and the vector specific to negative contents:

$$\begin{array}{l} Response\;negativity=\frac{\vec{s}}{\left\|\vec{s}\right\|}\times \frac{{\vec{s}}^{-}}{\left\|{\vec{s}}^{-}\right\|}\\ \;=\sum_{1}^{i}\left[\left(\begin{array}{c} z_{1}\\ z_{2}\\ z_{3}\\ \ldots \\ z_{i} \end{array}\right)\times \left(\begin{array}{c} z_{1}^{-}\\ z_{2}^{-}\\ z_{3}^{-}\\ \ldots \\ z_{i}^{-} \end{array}\right)\right] \end{array}$$

Response positivity and negativity must always be interpreted together, with an extra focus on asymmetric combinations. In particular, a high positivity and a low negativity highlight strong socially desirable response strategies; such results suggest that the individual has adequate representations of normative expectations, and that s/he intends to satisfy these expectations. On the contrary, a low positivity and a high negativity can result from faking bad, or could suggest a lack of conscience of social expectations, which is also of some interest for investigation. However, large differences between both indices systematically lower the validity of the assessment (Gendre and Capel, 2013).

## Expressing results in an absolute metric

The second issue that FMT purports to address is the problem of arbitrary and relative metrics. Of course, expressing psychological measures in standard scores has unquestionable advantages and is still available in FMT. Besides, as previously stated, FMT provides measures in an absolute metric, which makes the advantages of IRT available in FMT. In particular, comparisons of scores in different measures become possible, since they are eventually expressed in the same absolute metric. The advantages include being able to compare factor scores, item scores, or even individuals, because they can be represented in the same multidimensional measurement space.

Nonetheless, the metric used in FMT has psychological meaning, which is a quality that the IRT-based metric does not have. Indeed, in the correlation-based metric in which factor scores are expressed, the 0 has a specific signification. It indicates neutrality. Moreover, such scores can eventually be expressed on the Likert-type scale that has been used for the items, which makes them very easy to understand. For example, for a test measuring vocational interests using a 5-point Likert-type scale (1 = *strong disinterest*, 5 = *strong interest*), one must merely multiply the correlation by 2 and add 3 to express scores on the very same metric as the response space. In that metric, a 3 corresponds to indecision or neutrality, which effectively gives a specific signification to the 0 in the correlational metric. Such a metric can be very useful for psychological practice: Indeed, it alleviates most of important sources of confusion related to tests feedbacks by providing scores that can be compared with other ones and with one's original answers to the questionnaire.

## Detecting and excluding invalid results

By employing new control indices, the functional method makes detecting invalid results possible, which is of great interest for both individual assessment and even globally for test calibration; yet, some guidelines need to be formulated in order to have a standard procedure of detecting and excluding invalid results. Control indices provide information regarding response patterns; nonetheless, the possibility to rely on control indices does not alleviate the question of other sources of biases (e.g., biases related to data collection, which are beyond the scope of this paper).

Because the definition of acceptable conditions largely depends on the test used, proposing absolute values for each index is of little relevance; this is why precise values will only be provided regarding response coherence, reliability, modality, and normativity. They can be summarized in the following questions:

1. Do the results meet basic technical requirements for functional modeling? In order to check this point, two sub-questions should be asked:
  - Is the response variability high enough? As previously stated, results with little variance result from information retention; they are thus totally unusable.
  - Is the response level well within the norms? If not, this may result from extreme response biases (e.g., acquiescence or opposition), and cause problems regarding data homoscedasticity; this may lead to calculable but deceptive results.
2. Can the response strategy be regarded as valid? In order to determine this is so, three questions needs to be formulated:
  - Is the response reliability high enough? If not, this suggests either random answering, carelessness, inattention, or comprehension problems. In any case, this will lead to misleading results that should not be interpreted. According to Capel (2009), response reliability should be over 0.70 in order to consider test results reliable; response reliabilities between 0.50 and 0.70 highlight questionable results; and response reliability under 0.50 indicates unreliable results.
  - Is the response coherence high enough? If not, this corresponds to unusual or even abnormal response strategies; this can be caused by problems in understanding the items, but can also be related to immaturity (Capel, 2009). Because response coherence is equal to the root square of an *R*^2^ coefficient, acceptable values depend largely of the number of retained factors. Yet, for a 5-factor personality inventory, Gendre and Capel (2013) propose to consider response coherences lower than 0.40 as unsatisfactory. In such cases, this should be discussed with the subject in order to identify whether s/he understood the items; in this specific case, results should not be interpreted.
  - Is the response coherence too high? For a 5-factor personality inventory, values above 0.75 should be considered as too high (Gendre and Capel, 2013). If yes, this indicates that one is describing an expected profile rather than oneself; in other words, the person is faking so that the results are no longer personally representative. Such cases should be analyzed in regard with positivity and negativity in particular.
3. Is there any other specific issue related to response strategy? An answer can be given by the four last indices, specifically the articulation between response normativity and response modality, and between response positivity and negativity. Two kinds of questions should be asked:
  - Is the response normativity negative? The response modality, when based on Cohen's weighted kappa, is likely to be slightly negative; thus, a supplementary question would be: Is it under −0.50? If so, this questions how one has understood the instructions; indeed, such results suggest that the person might have reversed his or her answers by mistake, so that s/he agreed with items that s/he was supposed to disagree with, and vice versa. Such a situation is clearly problematic, because it results in valid and relevant, yet aberrant, information, which could remain thus far undetected. In clinical practice, this should be quickly checked with the participant; in such cases, results might be usable after re-reversing the participant's answers.
  - Is there a balance between response positivity and negativity? If not, different cases related to socially desirable responding might be met. When one has a high positivity and a low negativity, this suggests that one's response are refined in order to show desirable traits on one hand, and to hide undesirable traits on the other hand. This corresponds to forms of faking that could vary in terms of intensity. Cases in which one has underlined one's undesirable aspects or weak points require further discussion; in clinical practice, in particular, they should be questioned seriously. Indeed, these answers could either suggest that the person is not able to recognize socially-expected attitudes, or may result from severe self-denigration (e.g., self-loathing as a symptom of depression).

**Figure 1:**
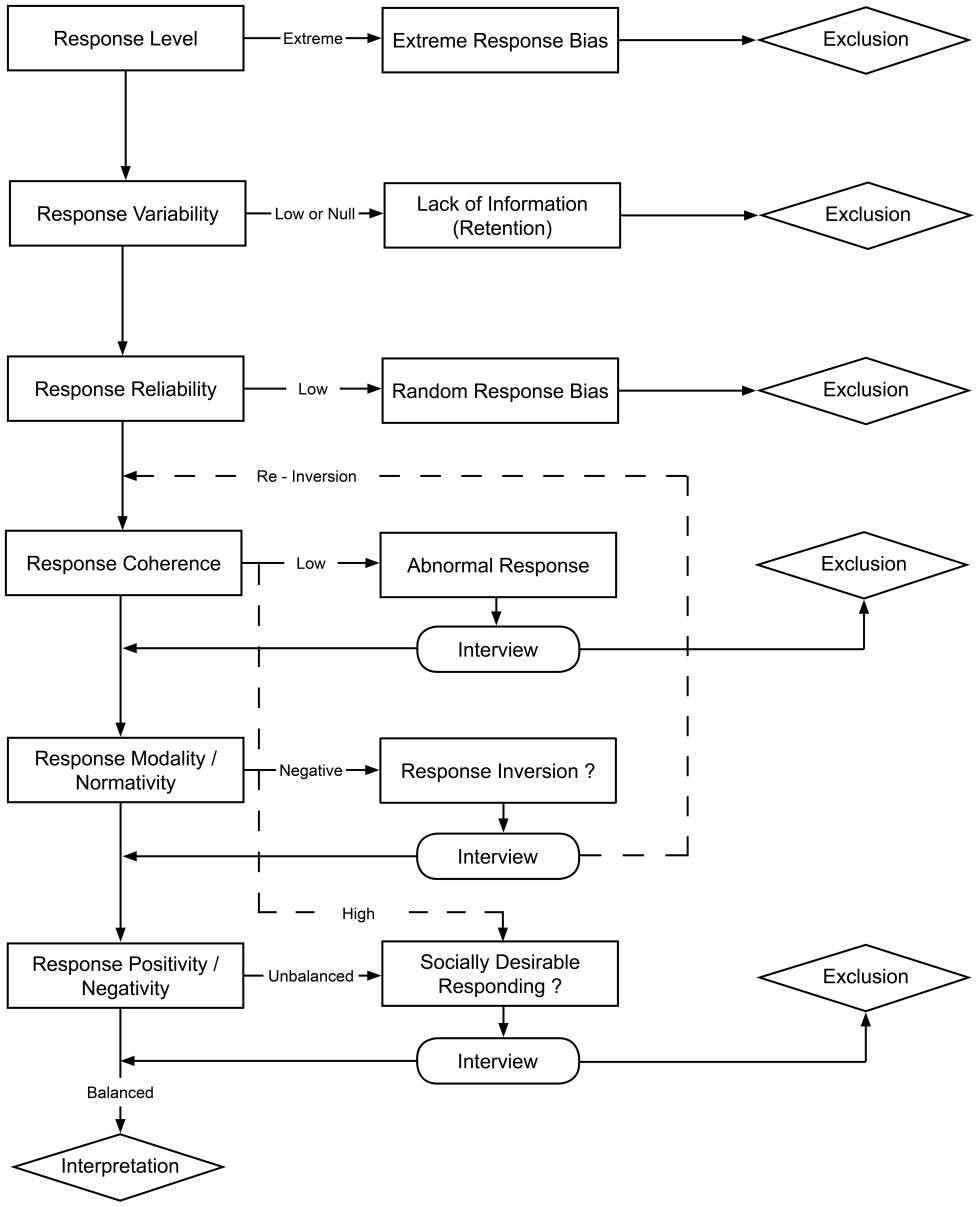
**A decision flowchart for determining whether one's test results are interpretable or not**.

Of course, this procedure does not alleviate the question of other problems impacting the validity or the representativeness of the results. Nevertheless, test results that meet all of the above requirements are likely to be valid and interpretable regarding the most detrimental response biases known so far. Figure 1 summarizes this standard procedure in a decision flowchart.

A remaining question concerns what to do with results that do not meet the criteria. In clinical practice, such results are simply unusable and should be excluded; thus, the clinician must find another way to assess scores from participants with invalid responses. In research, invalid response sets cannot simply be excluded: They would not be missing at random. Response biases are unlikely to be met uniformly among the population; they are part of normal responding, and they are related to other factors like intelligence (Gudjonsson, 1990) and socioeconomic status (Meisenberg and Williams, 2008). This is why excluding biased results can cause problems regarding representativeness, and should be considered with extreme caution.

## Comparing FMT, CTT, and IRT

Understanding FMT basics and features allows us to better discuss the differences between FMT, CTT, and IRT. A summary of this comparison between the three approaches is given in Table 2:

**Table 2:** **A comparison of CCT, IRT, and FMT characteristics**.

	**CTT**	**IRT**	**FMT**
The standard error of measurement is consistent across scores, but differs across populations.	✓		✓
The standard error of measurement varies across scores, but is consistent across populations.		✓	
Shorter tests can be as reliable as longer tests.		✓	✓
The comparison of test scores across different forms does not depend on test parallelism.		✓	✓
Unbiased estimates of item characteristics can be calculated using non-representative samples.		✓	
Trait scores are measured by comparison of distances from the items.		✓	(✓)
Trait scores are measured by correlations between the vector of strategy and the items' characteristics.			✓
Individuals, items, and factor scores are expressed in the same metric and are therefore comparable.		✓	✓
Scores are expressed on a continuous metric.		✓	✓
The scores are expressed on an absolute metric.		✓	✓
The absolute metric proposed carries a psychological meaning.			✓
The method is specifically designed for multidimensional tests with polytomous items.	✓		✓
Each item is assumed to be correlated to every factor.			✓
The prediction of other test scores is maximized by design.		✓	✓
Item-specific person-fit analyses can be conducted.		✓	✓
General person-fit adequacy can be measured.		✓	✓
Specific functional indices (i.e., coherence, reliability, positivity, negativity) can be calculated.			✓
Non-specific indices (i.e., level, variability, modality, normativity) can be calculated.	✓	✓	✓
Explicative hypotheses concerning person-fit inadequacy can be empirically supported by indices.			✓

*Note. ✓, theoretically assumed; (✓), theoretically assumed and applicable, and not yet exploited*.

Embretson's (1996) rules of measurement provide a good starting point for comparing FMT, CTT, and IRT. Regarding the standard error of measurement, FMT is comparable to CTT: The standard error of measurement differs across populations, but applies in every score. In FMT, the reliability of a test is less dependent on its length than in CTT. Indeed, in FMT, item multidimensionality is considered as a source of precision in calculating one's strategy that will eventually be used to calculate trait scores, whereas in CTT multidimensionality is considered a source of error used to calculate the trait score. Practically, the reliability in FMT relies on test length, but also on a number of other factors. Much like IRT, the comparison of test scores in FMT does not depend on test parallelism for two different reasons. First, the bisection method applies to response strategy; thus, in FMT it is the parallelism between half-test strategies that matters instead of the parallelism between test forms. And second, FMT is more like IRT in that all item-, trait- and person-related scores are expressed in the exact same metric, score equivalences can be ensured by design. Concerning the estimation of item characteristics, FMT relies on the same basics as CTT; thus, item characteristics can be calculated only on the basis of representative samples. Regarding trait score calculation in FMT, obtaining measures by comparing the distance from the items is theoretically feasible, yet not proposed; instead, trait scores are the correlations between the responses and the items' characteristics.

FMT also differs from CTT or IRT in several other ways. Unlike CTT, FMT uses a metric that is absolute and continuous (i.e., not the sum of ordinal scales) to express the characteristics of items, traits, and individuals. The main difference with IRT consists in the metrical base: In IRT, scores are expressed in logits, while in FMT they are expressed in correlations. Both approaches are very similar on this aspect, especially since both metrics result from the same mathematical foundation (i.e., correlations). Notwithstanding, when scores are expressed in the correlational metric, they have a psychological meaning. A correlation of 0 indicates neutrality, whereas positive correlations indicate that the person endorsed the item (or the trait); and negative correlations indicate that s/he rejected the item.

Like CTT, FMT is specifically designed for multidimensional tests with polytomous (i.e., Likert-type) items. Yet, FMT differs from CTT in the assumption that each item is correlated to each retained factor. By design, both IRT and FMT are optimal for predicting other test scores or outcomes. Indeed, in FMT, predictions rely on factors that are independent, which prevents them from overlapping in predictors.

Last, FMT differs from the other approaches in person-fit adequacy, which is not investigated by CTT. In both IRT and FMT, general and item-specific adequacy is estimated; FMT focuses on general adequacy by estimating response coherence, yet item-specific features are also possible. Furthermore, functional control indices using the common correlational metric make problematic response patterns more interpretable. In other words, CTT provides very little information about question-response strategy; IRT renders detecting problematic response patterns possible; and FMT proposes a systematic detection of problematic response strategies and provides information in order to interpret such problematic patterns.

## Further issues in functional modeling

As a new approach, FMT offers opportunities for various applications in psychological assessment. Here are some developments that have been or are currently undertaken:

– *Making FMT evidence-based*: One major issue is to provide clear evidences supporting the validity of the functional method, which includes the control indices as well. For the moment, different instruments have been developed and validated. Based on those, split-half reliability was calculated among the control indices (except response reliability, on which the bisection method is not applicable). Indices split-half reliability varies from 0.70 to 0.95, clearly sustaining their reliability (Gendre et al., 2011, 2012; Gendre and Capel, 2013). To provide evidence FMT should be applied to well-validated tests, and comparison between both classic and functional forms in terms of validity and reliability should be made.
– *Modelling and predicting other measures*: Because it is based on multiple regression modeling, another interesting feature of the functional method is the possibility of predicting various measures. Those comparisons can include answers to items (indicating how a perfectly coherent person would have answered an item, based on every other answer), or even to other scales that have been completed for concurrent validation. This point may be of great interest in measuring psychological traits that are altered as soon as one is aware that they are measured.
– *Identifying groups unable to provide valid responses*: Some subgroups of participants are more likely to provide invalid responses to self-rated tests and questionnaires (Meisenberg and Williams, 2008; Soto et al., 2008). Nonetheless, it is not clear who is concerned and how likely they are to provide invalid results. Continuing to use classic tests on them causes a stalemate. In both research and clinical practice, it consists of invalid data collection, resulting in a loss of time and money. Therefore, another important application would be to identify which subgroups of the population are unable to respond correctly (i.e., coherently, reliably, etc.). By determining who is at risk, it will be possible to develop adequate clinical or statistical alternatives.

Such issues highlight certain CTT standstills. Therefore, verifying how FMT effectively deals with them is an aim of major importance.

## Limitations

There are some limitations to this method. As a fully multivariate model, it takes every association between variables into account; this makes it a more realistic approach of complexity, but also makes it incompatible with some typical psychometrical methods of validation (e.g., calculating reliability using Cronbach's alpha or performing confirmatory factor analyses). In addition, calibration also requires large sample sizes in order to make a test sensitive enough to small factor loadings; if not, small factors are indeed likely to generate statistical noise instead of useful information.

## Conclusion

To conclude, the functional method is a new test theory in which scoring technique can be applied to Likert-type scales and other kinds of psychological questionnaires. FMT provides measures for multiple dimensions and expresses them in an absolute metric. Based on the same multivariate techniques as classic tests, it uses the same amount of extracted variance, but more precisely. It can be applied conjointly with classic scoring techniques, in order to provide information about how valid collected responses are, and which responses are not, and addresses some important sources of error in testing.

### Conflict of interest statement

The authors declare that the research was conducted in the absence of any commercial or financial relationships that could be construed as a potential conflict of interest.

## Footnotes

^1^PCA is used instead of factor analysis because the aim of the mathematical operations is to conserve the best summary of total variance, not to extract common variance.

^2^The term “strategy” refers to game theory; it does not mean that the person has the intention to answer to the test in a certain way.
