# Supplementary material for: Measuring individuals' response quality in self-administered psychological tests: an introduction to Gendre's functional method
Source: Front Psychol. 2015 May 15;6:629. doi: 10.3389/fpsyg.2015.00629 (PMC4470441; doi:10.3389/fpsyg.2015.00629)
Supplement: Supplementary file 1 [file DataSheet1.DOCX]

**Appendix 1: *A 13-point summary of the functional method***

1. Take a field of study that is wide and complex (e.g. personality, vocational interests, values, …) and is measured using self-rated questionnaires.
2. There is the **universe of the items** measuring this topic.
3. There is the **universe of the items of the test** that is a representative sample of the items measuring the topic (generally they can be summarized by 4 to 6 factors, with about 15 to 30 items per factor).
4. There are **characteristics of the items**, comprising the information carried by the items that is collected by a mix of three methods: rational (experts’ judgements), statistical (the factor structure of the test), and empirical (items’ concurrent validity).
5. There is the **measurement space** obtained by performing a factor analysis on the item characteristics, and reiterating the process until all communalities are 1. This space is orthonormal and hyperspherical; thereby, the items are **unit radius vectors** whose coordinates are equal to factor loadings.
6. Three kinds of scales could be expressed in the measurement space by unit radius vectors: **factors** resulting from factor analysis; **internal scales** centroid aggregates resulting from cluster analysis; and **external scales** predicted by the factors.
7. In addition to the measurement space, there is the **response space**, in which vectors containing individuals’ responses to the items expressed on a Likert-type scale (with 5 points or more) are represented. The vectors’ values can be ordinal, but are non-metric.
8. **One’s response vector is then metricized**. The metrication corresponds to the case with maximum multiple correlation between the response vector and the item vectors.
9. The functional method consists of modelling one’s response strategy using a **multiple**

**regression model predicting the response space by the measurement space**.

1. The multiple correlation and the beta weights resulting from the model correspond, respectively, to the **coherence of the response strategy** (i.e., response coherence) and the coordinates of the response strategy. The response strategy is normed and is thus a unit vector of the measurement space.
2. Beside the response coherence, different indices can be obtained using specific vectors of strategy (i.e., **response reliability**, **positivity**, and **negativity**).
3. The normed strategy is used to calculate the predicted item’s responses and the score to the scales. They represent a new form of scoring that relies on an absolute metric, which makes **intra-individual interpretation** possible.
4. Furthermore, inter-individual comparisons based on standardized scores are still possible. **Mixing intra-individual and inter-individual interpretations** results in a better approach to understanding individuals’ complex characteristics.
